# A preliminary checklist of the ants (Hymenoptera, Formicidae) of Andorra

**DOI:** 10.3897/zookeys.277.4684

**Published:** 2013-03-15

**Authors:** Abel Bernadou, Vincent Fourcassié, Xavier Espadaler

**Affiliations:** 1 Université de Toulouse, UPS, Centre de Recherches sur la Cognition Animale, 118 route de Narbonne, F-31062 Toulouse cedex 9, France; 2 CNRS, Centre de Recherches sur la Cognition Animale, 118 route de Narbonne, F-31062 Toulouse cedex 9, France; 3 Departament de Biologia Animal, de Biologia Vegetal i d’Ecologia, Facultat de Ciències, Universitat Autònoma de Barcelona, E-08193 Bellaterra, Spain; 4 Present address: University of Regensburg, Biologie I, Universitätsstraße 31, 93053 Regensburg, Germany

**Keywords:** Hymenoptera, Formicidae, checklist, new records, Andorra, Pyrenees

## Abstract

Within the last decade, checklists of the ant fauna of several European countries have been published or updated. Nevertheless, no ant checklists have hitherto been published for the principality of Andorra, a small landlocked country located in the eastern part of the Pyrenees. This work presents a critical list of the ant species of Andorra based on a review of the literature and on the biological material we collected during several field campaigns conducted in Andorra since the year 2005. Seventy-five species belonging to 21 genera of Formicidae were recorded. Nine species were recorded for the first time in Andorra: *Aphaenogaster gibbosa* (Latreille, 1798), *Camponotus lateralis* (Olivier, 1792), *Camponotus piceus* (Leach, 1825), *Formica exsecta* Nylander, 1846, *Lasius piliferus* Seifert, 1992, *Tapinoma madeirense* Forel, 1895, *Temnothorax lichtensteini* (Bondroit, 1918), *Temnothorax niger* (Forel, 1894), *Temnothorax nigriceps* (Mayr, 1855). The most speciose genera were *Formica* Linnaeus, 1758 and *Temnothorax* Forel, 1890 with 14 and 12 species, respectively. The ant fauna of Andorra is mostly dominated by Central European species (some are typical cold climate specialists); however species belonging to the Mediterranean ant fauna were also found. This can be explained by the particular geographic situation of Andorra which is characterized by a high mountain Mediterranean climate.

## Introduction

Over the last decade, ant taxonomy has experienced a renewal in Europe due to the description of new species (Seifert 2005), the revision of genera or species groups (e.g. Radchenko and Elmes 2003, Rigato 2011) and the publication of checklists of the ant fauna of several European countries (e.g. Austria: Steiner et al. 2003, Benelux: Boer 2010, Bulgaria: Lapeva-Gjonova et al. 2010, Croatia: Bračko 2006; Romania: Markó et al. 2006, Montenegro: Karaman 2009, Poland: Czechowski et al. 2012, Slovenia: Bračko 2007). This renewal is also due to the publication of excellent taxonomic keys for ant identification (e.g. Seifert 2007, Boer 2010, Radchenko and Elmes 2010), the development of Internet databases accessible on-line (e.g. antbase.org: antbase.org; Ant Genera of the World: www.antmacroecology.org; AntWeb: www.antweb.org), as well as to the use of molecular approaches that have helped to resolve old and debated taxonomical problems (Bernasconi et al. 2011).

**Figure 1.**
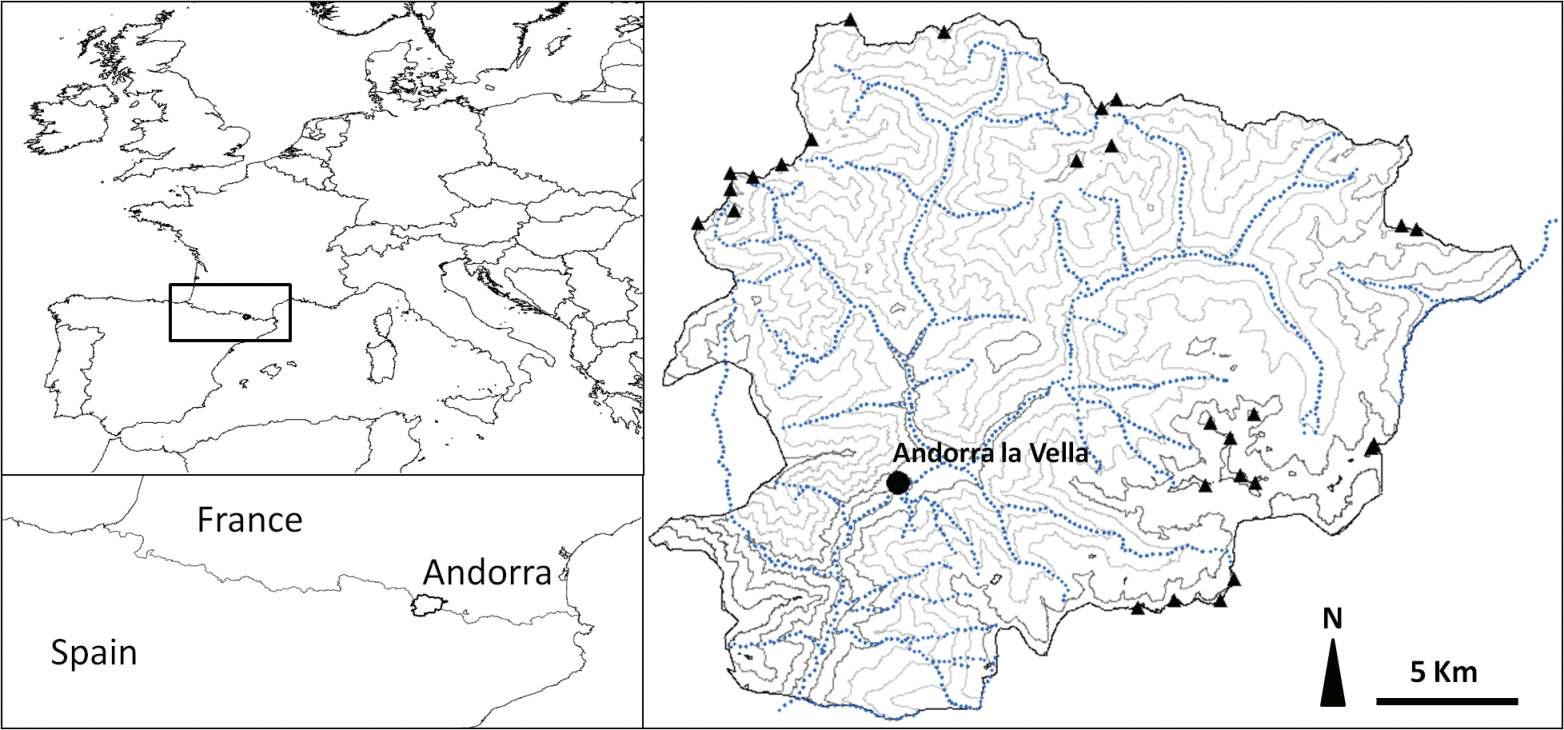
Map of Andorra (from light to dark grey: contour lines every 250 meters from low to high altitude, dotted lines: main Andorran rivers, triangles: peaks over 2800m).

Despite the acknowledged importance of mountain ecosystems (Kollmair et al. 2005) in conservation issues, little information is available in the literature on the ant diversity of European mountains. A case in point is the Pyrenees. These mountains have always been of great interest for naturalists because they are characterized by a relatively high rate of endemism of both animal (e.g. Deharveng 1996, Brown et al. 2009) and plant species (Villar and Dendaletche 1994). The Pyrenees are particularly interesting for myrmecologists because they are located in a climatic zone which covers three different regions from a biogeographical point of view: Alpine, Mediterranean and Atlantic. The principality of Andorra (Figure 1) is a small landlocked country located in the heart of the Pyrenees which is bordered by Spain and France and covers an area of approximately 468 km^2^ (Degage and Duro i Arajol 1998). Its relief mainly consists of fluvial valleys and rugged mountains spreading on an altitude ranging from 840 to 2942 m. Andorra has a high mountain Mediterranean climate, characterized by cold temperatures in winter (mean monthly temperature in January -2°C) and mild temperatures in summer (mean monthly temperature in July 19 °C), although extreme peaks of -20 °C at Ransol and 39 °C at Les Escaldes have been registred (Vilà-Valentí and Martín-Vide 1997). Although the ant fauna of France (Casevitz-Weulersse and Galkowski 2009) and Spain (Gómez 2012) are now relatively well known, the ant fauna of Andorra did not so far raise the interest of ant taxonomists. Except for some studies published in the grey literature or some scattered data collected during occasional samplings by Santschi (1919), Röszler (1937), Collingwood and Yarrow (1969), or Espadaler (1997) and Espadaler et al. (2008), there is little information available on the ant fauna of Andorra and no ant checklists for this country have been published to date. This paper presents a list of the ant species of Andorra based on a review of the literature and on material collected during several sampling campaigns conducted in Andorra since 2005.

## Methods

A preliminary checklist was assembled from a thorough and comprehensive review of the literature and from the information given by several databases available on the Internet (e.g. FORMIS 2011: www.ars.usda.gov/saa/cmave/ifahi/formis, ScienceDirect: www.sciencedirect.com, Google Scholar: www.scholar.google.com, etc.) using searching requests such as “Andorra + Formicidae”. This first checklist was compared with the material we collected during several sampling campaigns conducted in the last 8 years in different parts of Andorra: low and high altitude grasslands, low and high altitude forests, anthropized areas, etc Ants were searched on the ground and on vegetation; potential nesting sites were also inspected: dead wood, underneath of stones / bark, grass stems, acorns. Ants were collected by hand and were placed in plastic vials filled with 90° ethanol. Once in the laboratory, they were sorted and identified to the species level. All the material collected was identified by Dr. Xavier Espadaler. As no identification keys exist for the ants of Andorra, keys created for other Europeans countries (e.g. Czechowski et al. 2012, Seifert 2007) and for the taxonomic revisions of some ant genera (Seifert 1988, 1992, 2000, 2007) were used. Most of the material we collected is stored in the personal collection of Dr. Xavier Espadaler at the Department of Animal Biology, Plant Biology and Ecology, Autonomous University of Barcelona, Spain.

The final checklist was assembled based on every taxonomical/biodiversity papers related to Andorra as well as on other papers referring to data collected in Andorra (e.g. phylogeographical studies) and on the material collected by ourselves. The species list we present is arranged in alphabetic order by subfamily, genus and species. Nomenclature was checked following Bolton (2012). Only species with unambiguous taxonomic determinations were considered in the final checklist.

## Results

The ant species recorded in Andorra are listed in Table 1. The list contains 75 species distributed across 21 genera belonging to 4 subfamilies (Dolichoderinae, Formicinae, Myrmicinae, Ponerinae). The most speciose subfamily was Myrmicinae and the most speciose genus was *Formica* Linnaeus, 1758 with 36 and 14 species respectively. We included in our checklist the species *Tetramorium pyrenaeicum* Röszler, 1936. This species was first described in Andorra and elevated to species rank by Röszler (1951) (Güsten et al. 2006). However, because its morphological characters do not correspond to any European *Tetramorium* species currently described and because no biological material remains in their original depositories (Muzeul Brukenthal, Sibiu, Romania and Zoologisches Museum der Universität Hamburg, Germany), Güsten et al. (2006) consider that the validity of *Tetramorium pyrenaeicum* as a taxonomically distinct species remains controversial. Nonetheless, we chose to retain this species in our list because the possibility exists that it could actually be a taxonomically distinct species, such as *Tetramorium* *D* or *Tetramorium* *E*described by Schlick-Steiner et al. (2006). We also decided to include *Solenopsis* sp. Westwood, 1840 in our checklist. Because of the absence of revised taxonomic keys on this genus however we did not attempt to identify the *Solenopsis* specimen we found to the species level. We agree with Casevitz-Weulersse and Galkowski (2009) that a thorough review of this genus based on new material would be necessary to clarify its situation in Europe. Indeed, between 1949 and 1977 Bernard (see Casevitz-Weulersse and Galkowski 2009) described many new species related to *Solenopsis fugax* (Latreille, 1798) using characters that turned out to be variable and irrelevant to correctly identify the different species of this genus. Therefore, we decided to only mention the presence of the *Solenopsis* genus in Andorra. Finally, *Monomorium pharaonis* (Linnaeus, 1758) was excluded from our checklist. In fact, this species was erroneously reported as present in Andorra by Passera (1994) citing Eichler (1978) as a source (see Wetterer 2010). In absence of new data on this species, we decided to exclude it from our list.

After comparing our material with the data available in the literature, we found that 9 species were new to Andorra:

*Aphaenogaster gibbosa* (Latreille, 1798), Sant Julià de Lòria: Bordes de la Juberrussa (42°26.41'N; 1°28.81'E - 950 m a.s.l), 15.VII.2007, leg. A. Bernadou, det. X. Espadaler, workers collected in a nest under a stone.

*Camponotus lateralis* (Olivier, 1792), Sant Julià de Lòria: Borda del Sabater (42°26.75'N; 1°28.83'E - 870 m a.s.l), 15.VII.2007, leg. A. Bernadou, det. X. Espadaler, workers collected.

*Camponotus piceus* (Leach, 1825), Sant Julià de Lòria: Coll de Jou, carretera de Fontaneda (42°27.50'N; 1°29.00'E - 1100 m a.s.l), 02.VIII.2008, leg. A. Bernadou, det. X. Espadaler, workers collected.

*Formica exsecta* Nylander, 1846, refugi de Sorteny (42°37.45'N; 1°34.56'E - 2100 m a.s.l.), 21.IX.2011, leg. det. X. Espadaler, workers collected.

*Lasius piliferus* Seifert, 1992, Sant Julià de Lòria: Coll de Jou, carretera de Fontaneda (42°27.50'N; 1°29.00'E - 1100 m a.s.l), 02.VIII.2008, leg. A. Bernadou, det. X. Espadaler, workers collected.

*Tapinoma madeirense* Forel, 1895, Sant Julià de Lòria: Coll de Jou, carretera de Fontaneda (42°27.50'N; 1°29.00'E - 1100 m a.s.l), 02.VIII.2008, leg. A. Bernadou, det. X. Espadaler, workers collected.

*Temnothorax lichtensteini* (Bondroit, 1918), Sant Julià de Lòria: Coll de Jou, carretera de Fontaneda (42°27.50'N; 1°29.00'E - 1100 m a.s.l), 02.VIII.2008, leg. A. Bernadou, det. X. Espadaler, workers collected.

*Temnothorax niger* (Forel, 1894), Sant Julià de Lòria: Coll de Jou, carretera de Fontaneda (42°27.50'N; 1°29.00'E - 1100 m a.s.l), 02.VIII.2008, leg. A. Bernadou, det. X. Espadaler, workers collected.

*Temnothorax nigriceps* (Mayr, 1855), Sant Julià de Lòria: Coll de Jou, carretera de Fontaneda (42°27.50'N; 1°29.00'E - 1100 m a.s.l), 02.VIII.2008, leg. A. Bernadou, det. X. Espadaler, workers collected.

**Table 1.** Checklist of the ant species of Andorra. The list is arranged alphabetically by subfamily, genus and species. Species names in bold characters refer to species recorded for the first time in Andorra. Bibliographic references are as follows: a = Bernadou et al. 2006a; b = Bernadou et al. 2006b; c = Espadaler et al. 2008 *Hypoponera eduardi* was misidentified with *Hypoponera punctatissima* in this study (Espadaler, pers. com.); d = Bernadou et al. 2010; e = Bernadou 2009; f = Seifert 1992; g = Santschi 1919; h = Espadaler 1997; i = Collingwood and Yarrow 1969; j = Leppänen et al. 2011; k = Cournault and Aron 2009; l = Bagherian et al. 2012; m = Röszler 1951.source

**Subfamilies**	**Scientific valid name**		**References**
DOLICHODERINAE	*Dolichoderus quadripunctatus*	(Linnaeus, 1771)	c
	*Tapinoma erraticum*	(Latreille, 1798)	a, b, c, e, k
	***Tapinoma madeirense***	Forel, 1895	
FORMICINAE	*Camponotus aethiops*	(Latreille, 1798)	c
	*Camponotus cruentatus*	(Latreille, 1802)	c
	*Camponotus herculeanus*	(Linnaeus, 1758)	a, b, e
	***Camponotus lateralis***	(Olivier, 1792)	
	*Camponotus ligniperda*	(Latreille, 1802)	a, b, c, e, i
	***Camponotus piceus***	(Leach, 1825)	
	*Camponotus truncatus*	(Spinola, 1808)	c
	*Formica decipiens*	Bondroit, 1918	a, b, e
	***Formica exsecta***	Nylander, 1846	
	*Formica foreli*	Bondroit, 1918	a, b, e
	*Formica frontalis*	Santschi, 1919	a, b, e
	*Formica fusca*	Linnaeus, 1758	a, b, c, e, g
	*Formica gerardi*	Bondroit, 1917	c
	*Formica lemani*	Bondroit, 1917	a, b, e
	*Formica lugubris*	Zetterstedt, 1838	a, b, e, i
	*Formica picea*	Nylander, 1846	a, b, e
	*Formica pratensis*	Retzius, 1783	a, b, e
	*Formica pressilabris*	Nylander, 1846	a, b, e
	*Formica rufa*	Linnaeus, 1761	a, b, e, g
	*Formica rufibarbis*	Fabricius, 1793	a, b, c, e, i
	*Formica sanguinea*	Latreille, 1798	a, b, e
	*Lasius alienus*	(Förster, 1850)	a, b, c, e
	*Lasius brunneus*	(Latreille, 1798)	c, e
	*Lasius distinguendus*	(Emery, 1916)	c, h
	*Lasius flavus*	(Fabricius, 1782)	a, b, e
	*Lasius fuliginosus*	(Latreille, 1798)	e
	*Lasius grandis*	Forel, 1909	a, b, c, e, f
	*Lasius mixtus*	(Nylander, 1846)	a, b, c, e
	*Lasius niger*	(Linnaeus, 1758)	i
	*Lasius paralienus*	Seifert, 1992	d, e
	***Lasius piliferus***	Seifert, 1992	
	*Lasius platythorax*	Seifert, 1991	e
	*Plagiolepis pygmaea*	(Latreille, 1798)	c
	*Plagiolepis xene*	Stärcke, 1936	c
MYRMICINAE	***Aphaenogaster gibbosa***	(Latreille, 1798)	
	*Aphaenogaster subterranea*	(Latreille, 1798)	c
	*Crematogaster scutellaris*	(Olivier, 1791)	c
	*Leptothorax acervorum*	(Fabricius, 1793)	a, b, e
	*Leptothorax muscorum*	(Nylander, 1846)	a, b, e
	*Messor structor*	(Latreille, 1798)	c
	*Myrmecina graminicola*	(Latreille, 1802)	c
	*Myrmica lobulicornis*	Nylander, 1857	a, b, e
	*Myrmica rubra*	(Linnaeus, 1758)	a, b, e, j
	*Myrmica ruginodis*	Nylander, 1846	a, b, e, i
	*Myrmica sabuleti*	Meinert, 1861	a, b, e
	*Myrmica scabrinodis*	Nylander, 1846	a, b, e, l
	*Myrmica schencki*	Emery, 1895	a, b, e
	*Myrmica specioides*	Bondroit, 1918	a, b, c, e
	*Myrmica spinosior*	Santschi, 1931	c
	*Myrmica sulcinodis*	Nylander, 1846	a, b, e
	*Myrmica wesmaeli*	Bondroit, 1918	a, b, e
	*Pheidole pallidula*	(Nylander, 1849)	c
	*Pyramica tenuipilis*	(Emery, 1915)	c, h
	*Solenopsis* sp.	Westwood, 1840	c
	*Stenamma striatulum*	Emery, 1895	c, h
	*Strongylognathus testaceus*	(Schenck, 1852)	a, b, e
	*Temnothorax affinis*	(Mayr, 1855)	a, b, c, e
	*Temnothorax gredosi*	(Espadaler & Collingwood, 1982)	a, b, e
	*Temnothorax kraussei*	(Emery, 1916)	c, h
	***Temnothorax lichtensteini***	(Bondroit, 1918)	
	*Temnothorax nadigi*	(Kutter, 1925)	e
	***Temnothorax niger***	(Forel, 1894)	
	***Temnothorax nigriceps***	(Mayr, 1855)	
	*Temnothorax nylanderi*	(Förster, 1850)	a, b, e
	*Temnothorax parvulus*	(Schenck, 1852)	e
	*Temnothorax rabaudi*	(Bondroit, 1918)	c
	*Temnothorax tuberum*	(Fabricius, 1775)	a, b, e
	*Temnothorax unifasciatus*	(Latreille, 1798)	a, b, c, e
	*Tetramorium impurum*	(Förster, 1850)	a, b, c, e, h
	*Tetramorium pyrenaeicum*	Röszler, 1936	m
PONERINAE	*Hypoponera punctatissima*	(Roger, 1859)	c, h
	*Ponera coarctata*	(Latreille, 1802)	c

## Discussion

With 75 species recorded, the ant fauna of Andorra can be considered as highly diverse, especially in view of the size of the country (Figure 1). The number of ant species collected represents more than one third of the number of species found in France (213 species, see Casevitz-Weulersse and Galkowski 2009) and about a quarter of the total number of species recorded in the Iberian Peninsula (299 species, Gómez 2012). When considered at the scale of the Pyrenees, Andorra contains about 88% of the ant species recorded in these mountains above an altitude of 1,000 m (about 85 species, Espadaler 1979, updated). Based on these results, we consider this first checklist as satisfactory. Nevertheless, we suspect that the total number of species could actually be somewhat higher for two reasons. First, we found very few parasitic species and one can imagine that they could probably be found with a higher sampling effort. And second, based on what is known from the ant fauna of France and Spain, some genera can be expected to be richer (e.g. *Temnothorax* Forel, 1890, *Camponotus* Mayr, 1861).

What could be the causes of the ant species richness observed in Andorra? Our data are interesting to compare with those obtained by Iserbyt et al. (2008) in a study of Pyrenean bumblebees in the Eyne valley, a small valley located in the eastern part of the Pyrenees. The number of species of bumblebees reported in this valley corresponds to 72% of the bumblebee species found in continental France. The authors explain this species richness by the high diversity of plants and habitat found in the Eyne valley. The same ecological factors probably contribute to the high ant biodiversity recorded for Andorra. In fact, with an altitudinal range of 2100 m condensed on a strong vertical gradient (Figure 1), Andorra presents a great diversity of microclimates and vegetation (e.g. sclerophyllous forest, mixed deciduous forests or mountain pine forests) that are potentially able to promote ant diversity. In addition to its relief, Andorra - because of its geographic situation on the south side of the Pyrenees - has a Mediterranean mountain climate (Degage and Duro i Arajol 1998). As a consequence, boreal ant species (e.g. *Camponotus herculeanus* (Linnaeus, 1758)) are listed jointly with species belonging to the Mediterranean fauna (e.g. *Camponotus cruentatus* (Latreille, 1802)). Such results have already been reported in other studies investigating the diversity of other insect orders in Andorra, e.g. Heteroptera (Gessé et al. 1994) and Sphecidae (González et al. 2000). The fact that this region is influenced by both an Alpine and a Mediterranean climate gives an additional interest to the study of the natural heritage of Andorra and makes the Pyrenees an ideal natural laboratory to study the influence of ecological factors on arthropod species diversity.
